# Barriers and Facilitators for the Implementation of Primary Prevention and Health Promotion Activities in Primary Care: A Synthesis through Meta-Ethnography

**DOI:** 10.1371/journal.pone.0089554

**Published:** 2014-02-28

**Authors:** Maria Rubio-Valera, Mariona Pons-Vigués, María Martínez-Andrés, Patricia Moreno-Peral, Anna Berenguera, Ana Fernández

**Affiliations:** 1 Research and Development Unit, Fundació Sant Joan de Déu, Esplugues de Llobregat, Barcelona, Spain; 2 Spanish Research Network on Preventative Activities and Health Promotion in Primary Care (RedIAPP), Spain; 3 Research Department, Institut Universitari d'Investigació en Atenció Primària Jordi Gol (IDIAP Jordi Gol), Barcelona, Spain; 4 Departamento de Psicología clínica y de la Salud, Universitat Autònoma de Barcelona, Bellaterra, Spain; 5 Social and Health Care Research Center, University of Castilla-La Mancha, Cuenca, Spain; 6 Research Unit, Distrito Sanitario Malaga, Fundación Pública Andaluza para la Investigación de Málaga en Biomedicina y Salud (IMABIS Foundation), Málaga, Spain; 7 Centre for Disability Research and Policy, Faculty of Health Sciences, The University of Sydney, Sydney, Australia; University of Missouri Kansas CIty School of Medicine, United States of America

## Abstract

**Background:**

Evidence supports the implementation of primary prevention and health promotion (PP&HP) activities but primary care (PC) professionals show resistance to implementing these activities. The aim was to synthesize the available qualitative research on barriers and facilitators identified by PC physicians and nurses in the implementation of PP&HP in adults.

**Methods and Findings:**

A systematic search of three databases was conducted and supported by manual searches. The 35 articles included were translated into each other and a new interpretation of the concepts extracted was generated. The factors affecting the implementation of PP&HP activities in PC according to professionals were fitted into a five-level ecological model: intrapersonal factors, interpersonal processes, institutional factors, community factors and public policy. At the intrapersonal level we find professionals' beliefs about PP&HP, experiences, skills and knowledge, and selfconcept. The attitudes and behavior towards PP&HP of patients, specialists, practice managers and colleagues (interpersonal factors) affect the feasibility of implementing PP&HP. Institutional level: PC is perceived as well-placed to implement PP&HP but workload, lack of time and referral resources, and the predominance of the biomedical model (which prioritizes disease treatment) hamper the implementation of PP&HP. The effectiveness of financial incentives and tools such as guidelines and alarms/reminders is conditioned by professionals' attitudes to them. Community factors include patients' social and cultural characteristics (religion, financial resources, etc.), local referral resources, mass-media messages and pharmaceutical industry campaigns, and the importance given to PP&HP in the curriculum in university. Finally, policies affect the distribution of resources, thus affecting the implementation of PP&HP.

**Conclusions:**

Research on barriers and facilitators in the implementation of PP&HP activities in multirisk management is scarce. The conceptual overview provided by this synthesis resulted in the development of practical recommendations for the design of PP&HP in PC. However, the effectiveness of these recommendations needs to be demonstrated.

## Introduction

Despite the evidence supporting the effectiveness and benefits of primary prevention and health-promotion (PP&HP) activities in reducing both the risk and incidence of health-related problems in a number of areas [1]–[4], these are still not standard practice in primary care [5].

Primary care professionals have regular contact with the vast majority of the population, learn about the patients' social situation, provide continuous care and have access to referral service resources within the healthcare system and through community [6]. These all place primary care professionals in a good position to readily conduct PP&HP both in at-risk patients and in the general population as part of the comprehensive care program [7]. However, primary care professionals show resistance to implementing these activities, citing barriers in clinical practice such as workload and lack of skills and knowledge, problems related to the professional-patient relationship and lack of confidence in the effectiveness of these interventions [8], [9].

Several qualitative studies have been conducted to gather data on primary care professionals' views on PP&HP but these have tended to focus on the prevention of specific diseases or the promotion of specific health activities or lifestyle-modification factors. Physicians and nurses in primary care are faced with patients with multiple lifestyle health risks and so encounter various barriers when implementing multi-strategy PP&HP activities, which are considered complex interventions. Furthermore, primary care is a complex system where patients and professionals' objectives may not always be in harmony and barriers in distinct disciplines can vary widely. If a preventive strategy is to be successfully implemented in primary care, as with any complex intervention, one of the first steps is to identify the major obstacles and strategies for optimum intervention implementation. Dissemination and implementation science also stress the importance of evaluating the barriers and facilitators for the translation of effective and efficient programs into practice [10]. The best approach to identifying barriers and facilitators in the development of an intervention, from the perspective of the agents that have to implement it, is the use of qualitative studies [11], [12].

Synthesis of the qualitative evidence on barriers and facilitators for PP&HP in primary care will provide researchers, decision-makers and health professionals with a global picture of the difficulties and opportunities that primary care professionals face when developing a primary preventive strategy.

The study objective was to synthesize the available qualitative research on barriers and facilitators identified by primary care physicians and nurses in the implementation of PP&HP in adults through meta-ethnography.

## Methods

For the qualitative synthesis, we used a meta-ethnographic approach to aggregate the information, re-interpret it and develop a fresh contribution to the literature. This approach was developed by Noblit and Hare [13], and adapted to health research by Britten and colleagues [14].

### Research Question

We searched for qualitative studies exploring physicians and nurses' perceptions regarding the implementation of primary prevention and health-promotion activities addressed to adults in a primary care context. The phenomena of interest were the factors (barriers and facilitators) that have an impact on the implementation of these activities.

### Study Search

Two reviewers (AF and MRV) independently searched three electronic databases: Pubmed (inception-October 2012), Web of Knowledge and CINHAL (inception-January 2013). The databases listed were searched using strategies designed to maximize sensitivity. These are detailed in Table S1. For the hand search, to include as much relevant information as possible, colleagues and team members were asked to suggest relevant papers they were aware of and the bibliographies of retrieved articles were checked for studies not identified in the original electronic search [15].

### Inclusion Criteria and Study Selection

Studies written in English or Spanish were included when they explored the perceptions of primary care physicians and nurses by using qualitative methods for both data collection and analysis. Studies using mixed methods were included if the qualitative findings were reported and discussed separately from the non-qualitative findings. The focus of the study had to be primary prevention of chronic conditions or health promotion (lifestyle changes). Studies focused on vaccines, children or secondary or tertiary prevention were excluded (e.g., treatment of alcohol addiction, prevention of recurrence, prevention of diabetes complications). Papers interviewing professionals from different health settings (e.g., specialists, homeopaths, and physiotherapists) where the specific discourse of the primary health care professionals could not be discerned were also excluded. Studies were excluded if the focus lacked sufficient relevance or if the data was not analyzed qualitatively.

Identified studies were screened, in duplicate (AF and MRV), by reviewing the title and published abstract. The final full-text review and selection was made in triplicate by the two reviewers that had conducted the searches, and an extra reviewer (MPV, MMA, PM or AB). In cases of disagreement, the six researchers reviewed the paper and reached agreement.

### Quality Appraisal

There is no absolute list of criteria for quality appraisal in qualitative research studies. The use of checklists for the evaluation of the quality of qualitative studies has been much criticized [16], [17] and there is a notable lack of consensus when categorizing papers according to different quality appraisal methods [18]. As in a previous synthesis [19], [20], quality was not numerically scored but discussed in terms of research coherence and taking the utility of findings into account [21]. Also considered were the appropriateness of the research design to the research question, the adequacy of the data collection procedures, the appropriateness and rigor of analysis and the presentation of primary data.

### Data Abstraction and Synthesis

Two reviewers independently extracted study characteristics (methodology and sampling characteristics) and the key findings of the studies included by using an abstraction form in which they differentiated between first-order constructs (views expressed by the professionals interviewed in the original studies) and second-order constructs (interpretations made by the original authors based on the views of the respondents). The abstraction form allowed the reviewers to include comments and personal interpretations of the data as well as ideas for the third-order constructs. When necessary, the corresponding authors of the original papers were contacted to obtain extra information (12 out of 18 authors contacted provided responses).

Papers were then read again in inverse chronological order (last published papers first) by AF and MRV who, taking into account the abstraction forms, completed a table where first and second-order findings were listed and grouped. As a starting point for extraction, we grouped and mapped the second-order information into concepts that followed a series of stages developed by the research team for the delivery of PP&HP in primary care (1-Assessment of risk and/or healthy lifestyles, 2-Motivational interview, 3-Education/Advice, 4-Follow-up, 5-Referral) which we considered to be affected by cross-cutting issues related to the patient and the practitioners at the Micro level and other factors at the Meso and Macro levels (factors associated with practice and the health system model, and cultural aspects). Since the original authors used various words to refer to the same interpretation of results, we translated the results of the papers into a common form by extracting the information piece by piece through a process of constant comparison. To achieve this, we listed the second-order information from the first paper taking special care to respect the authors' original terminology. Subsequently, we extracted the findings from the second study, grouping similar concepts and adding new original-author terms for the same category to the description of the category. When key concepts were related but not exactly the same, they were extracted separately but grouped together in the extraction grid.

The process was repeated with all the studies until they had all been translated into each other [14]. During the process of translation, new interpretations and relationships between concepts (third-order information) emerged and were recorded for subsequent consideration in the re-interpretation of the data. When all the studies had been translated and aggregated into the grid, it was reviewed by the authors that had not participated in the translation process and who had checked that the first and second-order information that they had extracted from the original work had been adequately considered and translated in the grid.

By using the synthesis of the first and second-order information, we then generated the third-order constructs [14]. For the third-order synthesis (the interpretation of interpretations), the concepts or factors and categories (groups of concepts) were refined and the relationships between categories of factors were re-organized producing modifications in the first series of stages. Several reconceptualizations of the findings were developed and refined, following a line-of-argument synthesis that became a model that was fitted to an Ecological Model [22]. This was carried out by AF and MRV and reviewed and discussed by all the authors.

The synthesis was externally audited from commencement to conclusion by a group of researchers from the “Qualitative Health Research Group” (led by Dr Vázquez ML) of the “Consorci de Salut i Social de Catalunya” as well as by Primary Care professionals and researchers from the Spanish “Research Network on Preventative Activities and Health Promotion in Primary Care” (RedIAPP).

## Results

### Studies Identified

**Figure 1:**
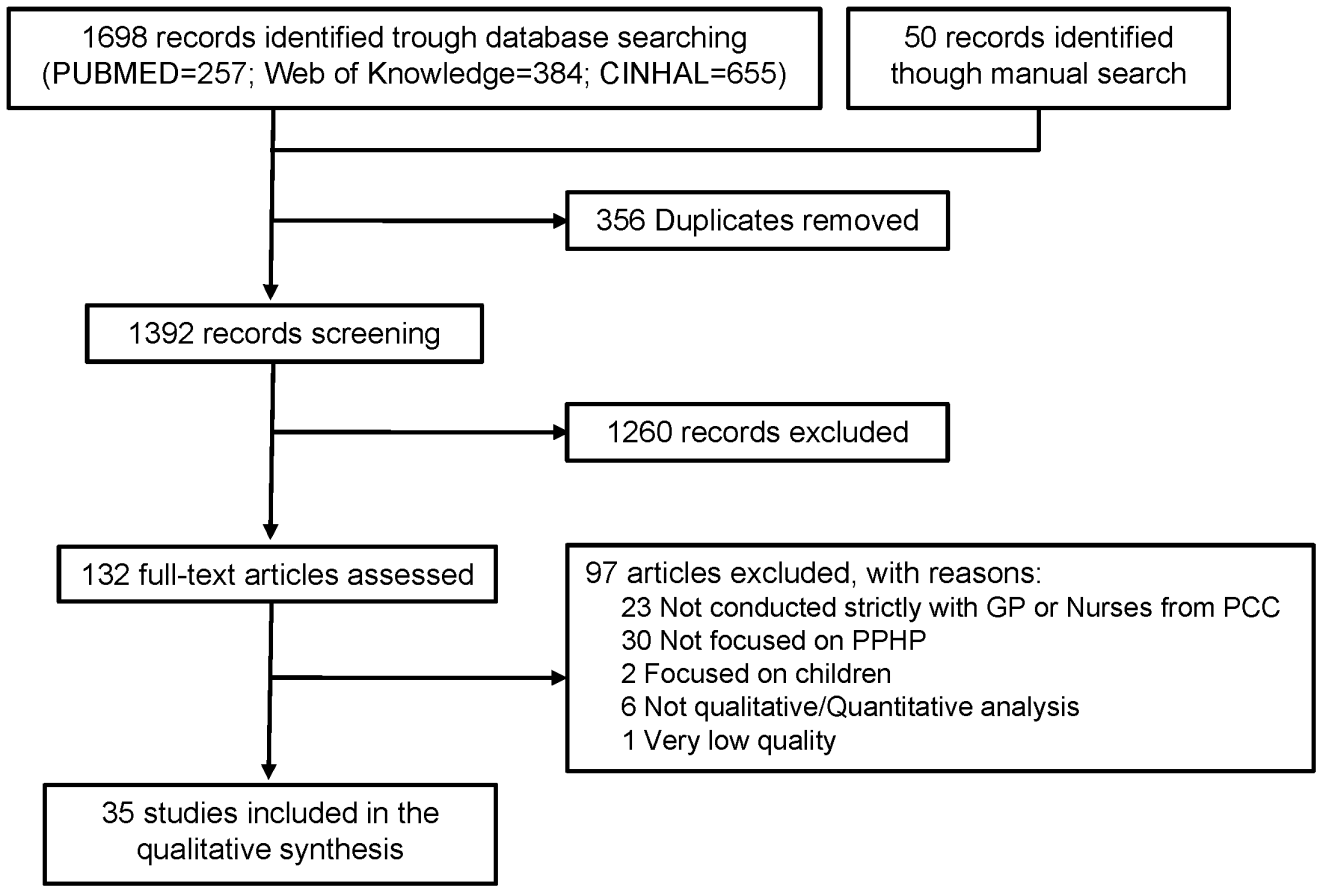
Flow-chart of the systematic review.

The database and manual search yielded 1,748 records and 35 were finally included in the synthesis (Fig. 1) [8], [9], [23]–[55]. Most of the studies interviewed GPs only (20), nurses only (5) or GPs and nurses (5) (see Table 1 for study characteristics). For data collection, the main methods used were semi-structured interviews and/or focus groups.

**Table 1:** Study characteristics.

	Study	Fieldwork year(s)	Country	Participants	Method of data collection	Aim (using original study wording)
1	Carlfjord 2012 [23]	2010	Sweden	9 GPs, 12 NPs, 6 nurse assistants, 3 allied professionals	Focus groups	To explore Primary Health Care staff perceptions of handling **lifestyle issues**, including the consultation situation as well as the perceived usefulness of a lifestyle computer-based tool.
2	Søndergaard 2012 [24]	2010	Denmark	16 GPs	Focus groups	To describe GPs' attitudes towards and concerns about providing **preventive health checks** and to describe their experiences with the health checks that they provide in daily practice.
3*	Badertscher 2012 [25]	2010	Switzerland	37 GPs	Focus groups	To assess attitudes, possible barriers to and facilitators of physicians to provide **health promotion** for the elderly.
4	Hernandez 2012 [26]	2010	USA	8 NP	Semi-structured interviews (narrative inquiry)	To explore the nurse practitioner experience with care for **prehypertensive** patients.
5	Gunther 2012 [27]	2009–2010	UK	7 GPs and 7 NPs	Semi-structured interviews	To reveal and describe the barriers and enablers to implementing NICE's recommendations for general practice teams on the management of **obesity** in adults (in the context of a local guideline implementation initiative).
6	Nolan 2012 [28]	2008–2009	UK	22 NPs	Semi-structured interview	To identify factors impacting on NPs' role adequacy and legitimacy regarding **obesity**.
7	Boase 2012 [29]	2005 and 2008	UK	28 Nurses	Semi-structured interviews and focus groups	To consider the perspectives of practice nurses in terms of how they approach communicating **cardiovascular risk** to patients within their clinical practice and the way that might influence how that information is received.
8	Kirkegaard 2012 [30]	–	Denmark	12 GPs	Focus groups	To explore GPs' experienced difficulties with decision making and risk communication with patients with high cholesterol and risk of cardiovascular disease.
9	Calderón 2011 [31]	2006	Spain	13 GPs	Focus groups	To gain an in-depth understanding of GPs' and patients' perceptions about **health promotion and prevention** in Primary Health Care and to define the areas that could be improved in future interventions.
10	Gale 2011 [32]	2003	UK	13 GPs	Single qualitative interview	To explore the attitudes of both patients and GPs towards **medication** for **primary prevention of cardiovascular disease** after they had received detailed information about cardiovscular disease risk and the absolute benefits of preventative medicine.
11	Müller-Riemenschneider 2010 [33]	2007–2008	Germany	24 GPs	Focus groups	To assess the use of and attitudes regarding the use of **risk scores for major chronic diseases** among GPs and to identify potential barriers to the use of risk scores for healthy adults in primary care.
12*	Walter 2010 [34]	2001–2003	German	32 GPs	Episodic interview	To identify and examine factors that promote and those that inhibit the implementation of **preventive care** for the elderly, as perceived by GPs, and to assess changes in physicians' attitudes toward preventive care throughout their careers.
13*	Heymann 2010 [35]	–	Israel	59 GPs; 14 residents specialising in family medicine; 12 geriatricians	Focus groups	To examine the barriers to **preventive health care** in the elderly among family physicians and to identify tools and devices that could help physicians augment these activities.
14	Ampt 2009 [36]	2007	Australia	15 GPs and 1 NP	Semi-structured interview	To identify the influences affecting GPs' choosing to screen and choosing to manage smoking, nutrition, alcohol consumption and physical activity **(SNAP)** **lifestyle risk factors**, as well as identify influences on screening and management when multiple SNAP factors exist.
15	Lambe 2009 [9]	2007	Republic of ireland	49 GPs and NP, 4 public health nurses, 1 social worker, 1 physiotherapist and 1 occupational therapist from the PCHC	Focus groups	To explore the views of primary health care practitioners about **behavioural risk factor management** in particular to the provision of **lifestyle counselling**. To identify barriers to behavioral risk factor management and to inform the development of a risk factor management tool kit for general practice.
16	Leverence 2007 [37]	2003–2004	USA	14 GPs, 7 peditricians, 9 NP and practice assitants	In depth interviews and focus groups	To examine the views of clinicians on **obesity** counseling and to compare these views to the recommendations of leading obesity guidelines.
17	Graham 2005 [38]	2004	UK	10 GPs and 2 NP	Semi-structured interview	To investigate the **exercise** referral process from the health professional's perspective, specifically examining perceived barriers to referral, priority given to an exercise referral scheme in day-to-day consultations, perceived importance of their role in the process and referring practices.
18	Puig Ribera 2005 [39]	2000–2001	Spain	18 GPs and 15 nurses	Semi-structured interview and focus group	To explore the experiences of doctors/nurses in promoting **physical activity** in their day-to-day professional lives.
19	Jacobsen 2005 [40]	2000	Denmark	5 GPs	Focus groups	To discover the views of Danish GPs on the possibility of intervening in their patients' **lifestyle** in general and on the obstacles to doing so, based on their experience of participating in a health promotion study.
20	Johannsson 2005 [41]	2000	Sweden	26 nurses	Focus groups	To identify under what circumstance nurses in primary care in Sweden are willing to engage in **alcohol** prevention and to what extent this is compatible with prevailing routines.
21	Williams 2004 [42]	2003	UK	21 GPs and 22 NPs	Focus groups	To explore the views of GPs and NPs about the detection and management of people at risk of developing **type 2 diabates**
22	van Steenkiste 2004 [43]	2000–2001	The Netherlands	15 GP	Observation and interview	To examine the barriers that prevent Gps from adopting the **cholesterol** guideline with its incorparated **risk tables**.
23	Hudon 2004 [44]	1997	Canada	35 GPs	Focus groups	To present the obstacles perceived by family physicians in Quebec concerning the integration of **prevention** into their routine practices.
24	Kedward 2003 [45]	2001	UK	26 GPs	Semi-structured interview	To identify GPs' views of the barriers to prescribing **statins**, their views of the use of statin guidelines, and their views of the barriers to, and successes in, implementation of **coronary heart disease prevention** in primary care.
25	Fulller 2003 [46]	–	UK	15 GPs	Semi-structured interview	To investigate the views of GPs and their patietns about **healthy eating** and the provision of healthy eating advice in general practice
26	Mirand 2002 [47]	2001	USA	12 GPs	Focus group	To identify conceptual themes that characterize primary care physician attitudes, deterrents, and practice environments regarding **preventive care** and, on the basis of the findings and to establish the conceptual framework of an intervention tool that will best meet the needs of primary care practices.
27	Beich 2002 [48]	2000	Denmark	24 GPs	Semi-structured interview and focus group	To explore the suitability of a screening based intervention for excessive **alcohol** use by describing the experiences of general practitioners who tried such an intervention in their everyday practice.
28	Lock 2002 [49]	1998	UK	24 nurses	Semi-structured interviews	To examine primary health care nurses' attitudes to **alcohol** intervention, including perceived barriers and facilitating factors, which influence their involvement in this area of work.
29	Coleman 2000 [50]	1995–1996	UK	42 GPs	Observation of patient-GP interaction and interview	To elicit, realte and interpret GPs' accounts of why they discuss **smoking** with some patients and not others.
30	Fairhust 1998 [8]	1997	UK	24 GP	Semi-structured interview	To explore how general practitioners have accessed and evaluated evidence from trials on the use of **statin lipid lowering drugs** and incorporated this evidence into their practice.
31	Makrides 1997 [51]	1996	Canada	31 GPs	Semi-structured interview and focus group	To explore the expectations of Nova Scotian physicians about their role in **prevention**, the obstacles they experience, and the mechanisms by which preventive care occurs.
32*	Kerse 1997 [52]	1995	Australia	20 GPs	Focus groups	To explore GPs' beliefs about **health promotion** for older people and attitudes towards educational strategies likely to improve practice in this area.
33	Swinburn 1997 [53]	–	New Zealand	25 GPs	Focus group	To assess the attitudes and perceptions of the GPs towards using the **green prescription**, and the feasibility of incorporating it into everyday practice.
34	Rush 1995 [54]	–	UK	24 GP	Semi-structured interview and focus group	To elucidate family physicians' motivations concerning early intervention for **alcohol** use and their perceived barriers to such interventions.
35	Williams 1994 [55]	1990–1992	UK	40 GPs	Semi-structured interview	To explore the GPs' perceptions of **coronary heart disease prevention**.

GPs = General Practitioners (physicians); NPs = Nurse practitioners.

*Studies focused on the elderly.

Most of the studies had been conducted in the UK (13), Denmark (4) and USA (3). Ten of the studies focused on primary prevention and/or health promotion in general terms while 13 of the studies focused on lifestyle risk factors including smoking, unhealthy eating, alcohol consumption and sedentary habits. The remaining studies focused on reduction of cardiovascular risk (8) (including use of lipid-lowering drugs), control of obesity (3) or prevention of type 2 diabetes (1).

### Quality Appraisal

The methods used in the studies were appropriate to answer the research questions. The analysis strategy, although poorly described in some of the studies, seemed appropriate, the presentation of the results was adequate and the conclusions of the studies were supported by the evidence presented. All the studies included showed coherence regarding research question and objectives, the methods used, the analysis strategy and the presentation of the results.

Many studies reported limited information on the theoretical context, the position of the researchers, the sampling strategy, the analysis strategy and the measures taken to ensure the rigor of the research and the validity of the findings. There was also limited information on the cultural and social context in which the study was conducted.

### Synthesis

**Figure 2:**
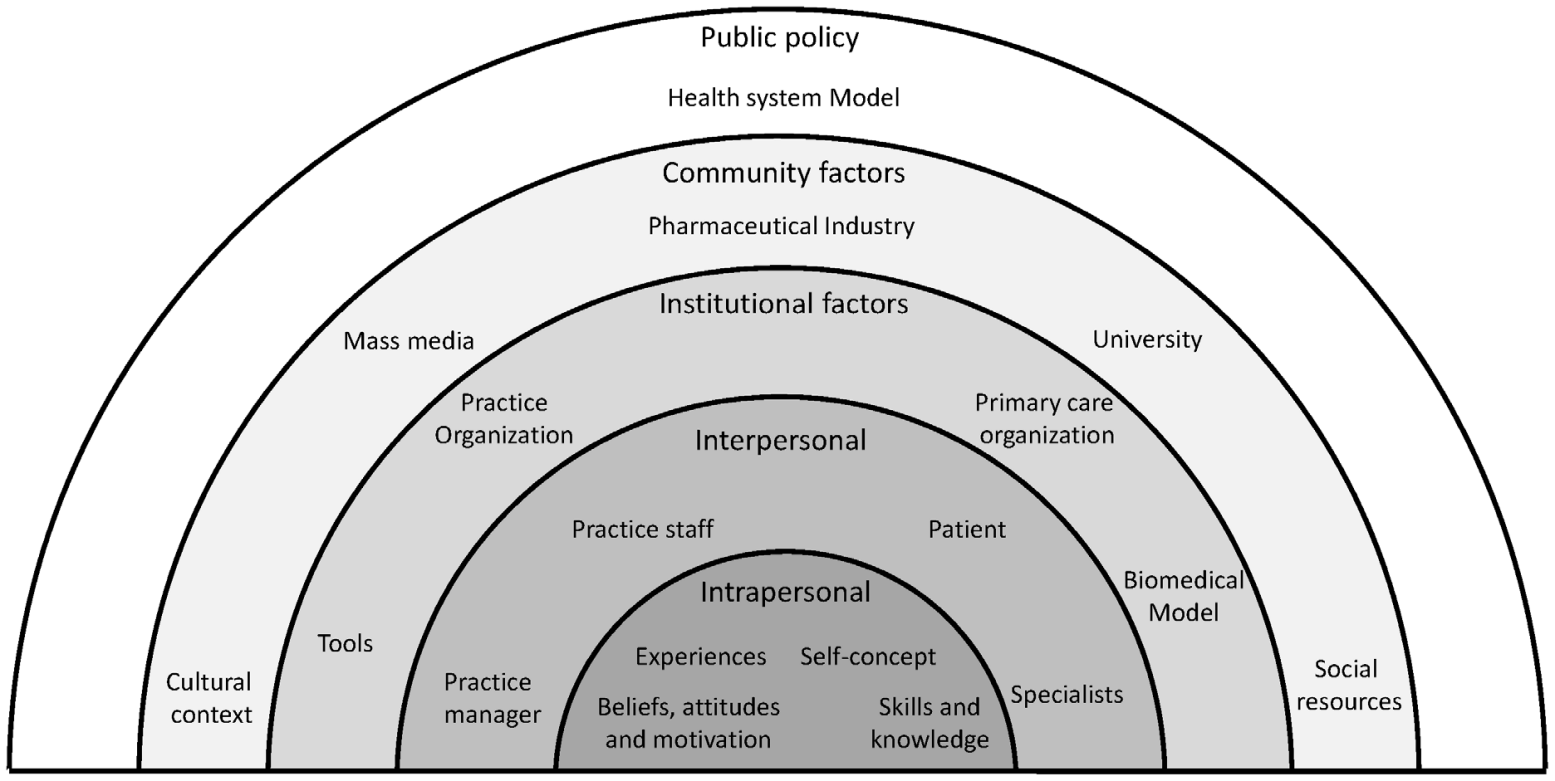
Ecological model of the factors affecting the implementation of PP&HP activities by primary care professionals.

A representation of the factors affecting the implementation of PP&HP activities in PC according to GPs and nurses is shown in Fig. 2. These third-order factors are arranged into five levels of influence on health professionals' behavior (multi-layer model that goes from micro to macro levels): intrapersonal factors, interpersonal processes, institutional factors, community factors and public policy.

Lower levels are affected by factors at the higher levels and factors at the same level can affect each other. The translation of the first and second-order constructs into third-order constructs and factors are summarized in Table 2 along with the paper from which first and second-order constructs are extracted.

**Table 2:** Translation of 1st and 2nd order constructs and interpretation through 3rd order constructs and sources.

3rd order FACTORS	3rd order constructs	2nd order constructs (translated)	Sources▵
INTRAPERSONAL factors	Experiences	Experiences dealing with the problem	8, 12, 28, 29
	Skills and knowledge	Evaluation of risk, communicative skills, motivational interview, counseling	1, 2, 4–8, 10–16, 19–28, 31–32
		Lack of knowledge about available resources for referral	3, 6,7, 9, 12, 14–16, 18, 20, 21, 23, 31, 32
		Lack of knowledge about available clinical guidelines	6, 11, 14, 15, 18, 26, 30
	Self-concept	Self-confidence	1, 5–7, 13, 15, 16, 20, 26–28, 31–33
		Professional as a role model or example to the patient (self-experience with the problem)	12, 18, 25, 28
	Beliefs	Risk is not a disease (primary care professionals' duty is to treat disease)	7, 18
		PP&HP is not effective/efficient	2, 3, 6–10, 12–19, 21, 22, 24, 25, 27, 30, 32, 33, 35
		PP&HP is (not) primary care professionals' duty/responsibility (professional perception and/or “obligation”)	1–3, 5, 6, 9, 12, 14–22, 25–28, 31–35
		PP&HP is utopian	9
		PP&HP only makes sense in high risk patients but not in general population	6, 16, 18–20, 25, 27, 30, 31, 33, 34
		Negative aspects of available guidelines (depersonalize, not adapted to local services, not looking beyond ticking-the-box, lack of consistency, unethical)	5–11, 18, 22–24, 28, 30, 34
		Negative aspects of risk assessment, use of risk scores (morality of risk calculation, risk police, personal circumstances not taken into account, do not contribute any new information)	7, 8, 11, 15, 19, 22, 23, 27, 31, 33
		Medicalization of life	11, 21, 22, 30
		Use of preventive drugs (Easier than changing unhealthy lifestyles)	10, 22, 24, 30
	Motivation	Professional interest in PP&HP	12–14, 17, 23, 27, 31
	Attitudes	For or against the implementation of PP&HP in primary care	1–3, 5, 6, 9, 12, 14–22, 25–28, 31–35
Interpersonal	Practice staff	Confidence in the colleagues at the Primary Care Health Center	1, 4, 14, 16, 22, 34
		“Champions”, active promoters	1, 18
	Patient	Characteristics of the patient: age (motivation increases with age), psychological comorbidity.	8, 12, 13, 29, 32
		Lack of patient resources (economic, social, educational, and temporal)	4, 12–14, 16, 18, 23, 31, 32, 35
		Lack of interest and adherence, denial of responsibility and lack of feedback	1, 4–7, 10, 12, 14–18, 21, 23–28, 31, 32, 35
		Silver bullet	23, 26
		Demanding patient/Consumer patient (active role requesting/expecting the service)	2, 11, 12, 14, 22, 32, 35
		Patient agenda	1, 7, 8, 11, 15, 18, 19, 23, 25–27, 29, 31
		Side effects of PP&HP, can have an impact on the patient-professional relationship	1–3, 6, 8, 10, 17, 19, 20, 24, 27–29, 32, 34, 35
	Practice manager	Management commitment to PP&HP	1, 6, 13, 18, 31
	Specialists	Contradictory advice/discourse, fragmentation of care	12, 22, 23
Institutional	Biomedical model	Prioritizes the treatment of the disease instead of PP&HP, few resources assigned to PP&HP	1, 3–5, 12, 13, 15, 17, 18, 21, 25, 26, 30, 32, 34, 35
	Primary care organization	Ideal setting for PP&HP: credibility, well placed, continuity of care (facilitates spontaneous follow-up)	3, 7, 9, 14, 15, 17–19, 22, 23, 25, 28, 33, 34
		Workload/Lack of time	1–7, 9, 11–18, 20–27, 29–33, 35
		Lack of financial incentives for the service or the professional (Quality Outcomes Framework or Direction by Objectives)	1, 3–5, 7, 10, 11–13, 15, 23, 26, 31–33
	Practice organization	Role clarification and organized teams inside the Primary Care Health Center for referral and/or follow-up	1, 3, 6, 7, 9, 13–15, 18, 20, 21, 24, 25, 31, 33, 35
		Inadequate space, office organization, insufficient storage for preventive drugs	24, 31
		Flexible booking system	31
	Tools	Guidelines for risk assessment and interventions (useful as threshold to start treatment)	1, 3, 7, 11, 19, 24, 28
		Reminders (computerized or otherwise), programmed campaigns of risk assessment/promotion of healthy lifestyles (i.e. physical activity trimester, alcohol trimester)	11, 14, 20, 22, 24, 26, 29, 31, 32, 34
		Tools for better management or referral (computerized tools, web pages, leaflets, green prescriptions, etc.)	1, 3, 6, 8, 13, 14, 23, 26, 28, 32–34
Community	Pharmaceutical industry	Promotes prescription of preventive drugs instead of lifestyles changes	18, 30
	University	Lack of focus and/or education and training on PP&HP and the necessary skills to develop them	3, 6, 7, 11–13, 15, 18–20, 23, 26–28, 31–34
	Social context and resources	Patients' social circumstances that limit the possible interventions/referral (e.g., dangerous neighborhood, lack of affordable resources)	9, 15, 16, 19
	Cultural context	Immigrant patients: Language barriers, lack of culturally appropriate materials, awareness of patients' cultural differences when providing advice.	6, 14, 24, 34
		No social interest in investing in the elderly	3, 28, 33
		Lay people's views about PP&HP (patients think is about being checked, importance of obesity, smoking, drinking as beneficial, drinking as social activity).	16, 23, 28, 34
	Mass media	Importance given to PP&HP; Influence of role models on the patient.	12, 13, 26, 28, 33, 35
		Social marketing campaigns that reinforce the message from primary care professionals.	34, 35
Public policy	Health system model	Public or private models influence investment, payment for follow-up, referral, etc.	*

*It is not state in a particular paper but emerged when translating the papers from different countries.

▵ The numbers correspond to the numbers of the 35 included in the review as they are presented in Table 2. Lower numbers indicate newere studies and vice versa.

#### Intrapersonal factors

At this level we found: professionals' beliefs about PP&HP [8], [9], [23]–[25], [27]–[29], [31]–[49], [51]–[55],their experiences in dealing with a particular risk factor or required lifestyle modification [33], [49], [50],appropriate skills and knowledge [8], [9], [23]–[29], [31]–[37], [39]–[49], [51]–[54], their motivation [34]–[36], [37], [44], [48], [51], their attitudes [9], [23]–[25], [27], [28], [31], [33], [35]–[43], [46]–[49], [51]–[55] and their self-concept (self-confidence in their capacities and personal experiences with the problem: e.g., a smoker physician dealing with tobacco cessation or an obese nurse dealing with nutrition recommendations) [9], [23], [27]–[29], [33], [34], [37], [39], [41], [45]–[49], [51]–[53]. The beliefs are related to the consideration of risk as a disease or not, the effectiveness and/or efficiency of PP&HP activities, negative aspects (side-effects) of risk assessment and the medicalization of life, the use of medication as a preventive strategy (e.g., statins for cardiovascular-risk reduction), questions about which patients could benefit and who should be responsible for these activities, etc. These beliefs, together with the other factors described, affect motivation and attitudes towards PP&HP.

Some PC professionals discuss PP&HP from a biomedical perspective [8], [9], [23], [25]–[27], [34], [35], [38], [39], [42], [45], [47], [52], [54], [55]. From this perspective, which gives little importance to social factors, the prevention of disease and the promotion of healthy lifestyles are omitted. The reduction of risk, which is not considered a disease itself, is seen by professionals as peripheral to their field of work (it is an educational task and the responsibility of the community or the Government). Some professionals in this position describe these activities as uninteresting or even dull, boring and tedious [42]. From this perspective, the use of preventive medication, which is easier to prescribe than lifestyle modification activities, is preferred.

On the other hand, the PC professionals that adopt a biopsychosocial perspective perceive PP&HP as an important part of their role and thus feel responsible for implementing these activities in practice. This is related to their position in terms of who should be considered responsible when implementing PP&HP interventions. Professionals who think that PP&HP activities should only be addressed to high-risk patients (thus with a higher probability of developing a disease) are more accepting of implementing them in PC. In contrast, if PP&HP is to be implemented in the whole population, the PC professionals will share the responsibility with schools, the community and the media, and will play a limited role in it. This holistic approach is seen as utopian by some PC professionals.

There are two factors that affect professionals' motivation, the patient and the health system. Even when professionals have a positive attitude towards PP&HP, if they feel the patient is not interested, or does not adhere to their recommendations, they feel frustration. PC professionals think that the health system expects them to conduct PP&HP activities. This can also prove frustrating if the self-concept is low and/or the resources available are perceived to be scarce. This can affect motivation, changing the attitude towards PP&HP and setting up a vicious circle.

#### Interpersonal factors

From the PC professionals' point of view, the attitudes and behavior towards PP&HP of patients [9], [23]–[29], [31]–[52], [54], [55], specialists [34], [43], [44], practice managers [23], [28], [35], [39], [51] and colleagues [23], [26], [36], [37], [39], [43], [54] affect the feasibility of implementing PP&HP in PC.

The relationship that is established with the patient is mediated by their characteristics, their expectations about what will happen in the consulting room (usually related to the approach to the specific problem that brought the patient to the PCHC), and their own personal and economic resources. When the professional considers that the patient is not interested or does not have the resources to implement the required changes, he or she may decide not to invest time in providing advice on PP&HP. In fact, the professionals prefer not to implement PP&HP when they are concerned about damaging the patient-physician relationship, for instance, in dealing with issues related to alcohol consumption when this is not the motive for the consultation.

Other members of the PCHC team can act as facilitators, for example, the “champions” (colleagues who are highly motivated to implement PP&HP activities). A further facilitator is that the practice manager is involved and interested in these activities. Confidence in the competence of other PCHC team members could be a factor which predisposes the professional to implement the activities. The lack of coordination between different levels of care, such as the contradiction between messages coming from specialists and PC, complicates the implementation of PP&HP through PC.

#### Institutional factors

Professionals perceive that the biomedical model, which prioritizes disease treatment rather than prevention, is predominant in their institutions [8], [9], [23], [25]–[27], [34], [35], [38], [39], [41], [46], [47], [52], [54], [55]. This affects the professionals' beliefs, as stated above (Intrapersonal factors), and the organization of the practice [45], [51]. Professionals perceive that this perspective leads to few resources being allocated to implementation of PP&HP. Workload, lack of time and lack of referral resources hamper the implementation of PP&HP [8], [9], [23]–[29], [31], [33]–[39], [41]–[47], [50], [51]–[53], [55]. On the other hand, professionals think that the primary health care setting is well placed and has the necessary credibility to implement PP&HP [9], [25], [29], [31], [36], [38]–[40], [43], [44], [46], [49], [53], [54]. A facilitator is a well-organized practice where everyone knows their role regarding PP&HP and which has referral services within the practice (e.g., nutrition service) [9], [23], [25], [28], [29], [31], [35], [36], [39], [41], [42], [45], [46], [51], [53], [55].

Financial incentives, such as management by objectives, which reinforce some strategies, are perceived as a facilitator in some cases. In others, they can be perceived as undermining clinical objectives by giving an incentive to provide interventions based on activities that are easy to measure, encouraging quantity rather than quality [32]. For instance, a management by objectives strategy that incentivizes reduction of the levels of some biological indicators can encourage the prescription of drugs to achieve a quick fix rather than implementing lifestyle changes.

Tools such as guidelines and alarms/reminders are seen as facilitators for PP&HP [23], [25], [28], [29], [33], [35], [36], [40], [41], [43]–[45], [47], [49], [50], [51]–[54]. However, the usefulness of these tools is limited by whether the professionals consider implementation necessary.

#### Community factors

According to the professionals, the social, cultural and community context where the patient-physician interaction occurs will affect the decisions that the professional makes in relation to the initiation and development of PP&HP activities [9], [25], [28], [31], [36], [37], [40], [44], [45], [49], [52], [53]. For instance, in deprived areas where the patients cannot afford the local resources they are referred to, PC professionals could decide not to assess lifestyles or risks. Also, professionals perceive the patients' cultural aspects (e.g., country of origin or religion) as a potential barrier if they think that they are in conflict with the potential interventions or if they are not aware of what these values might be. Citizens' views can also affect what the professional feels is feasible to do in PC. For instance, drinking advice may be in conflict with citizens' views about drinking as a social activity. This could be supported by mass-media messages reinforcing the idea that moderate drinking can be a healthy habit [34], [35], [47], [49], [53], [55]. Nevertheless, professionals believe that mass media campaigns can be a useful tool in reinforcing health promotion messages; as was shown with smoking cessation campaigns [54], [55].

Professionals think that the curriculum in university and the pharmaceutical industry have an impact on their behavior [9], [25], [28], [29], [33], [34], [35], [39]–[41], [44], [47]–[49], [51]–[54]. Lack of undergraduate training in PP&HP activities is perceived as a barrier. With regard to the pharmaceutical industry, professionals feel that they are the object of marketing campaigns that promote the use of drugs to prevent diseases. Professionals feel that they are motivated through incentives given by pharmaceutical companies to prescribe drugs even when they perceive that the relative benefit of using drugs in comparison with lifestyle changes is not supported by the evidence [38], [50].

#### Public policy

When extracting first and second-order constructs, the importance of the health system model emerged although it was not directly stated by the professionals interviewed. Socioeconomic and political context affects the distribution of resources as well as the position individuals or groups hold within societies. Although barriers and facilitators for PP&HP activities are very similar in private and public systems, they are generated by different mechanisms. For instance, in a Private Healthcare System, such as that in the USA, where patients must pay for each visit, professionals feel that patients will be unwilling to accept follow-up visits. In contrast, in National Health Systems where services are free at the point of use, such as in Spain or the UK, follow-up is hindered by workload and limited time per visit.

## Discussion

The present synthesis of 35 original qualitative papers illustrates physicians and nurses' perceptions about the difficulties that they face when implementing PP&HP activities in primary care. The appropriateness of conducting these activities in primary care is not, in general, discussed by these professionals. However, the level of implementation is recognized as being low. Factors affecting implementation were fitted into a five-level ecological model going from Micro to Macro factors (Intrapersonal, Interpersonal, Institutional, Community and Public policy). The majority of barriers cited by the professionals are considered external barriers beyond their control, although the lack of self-criticism expressed is remarkable, as has been pointed out by Hudon [44].

### Implications for Practice

If PP&HP activities are to be successfully implemented and maintained over time in primary care settings, a series of factors needs to be taken into account. Table 3 summarizes the practical implications of the results of the synthesis.

**Table 3:** Practical implications of the results of the synthesis.

INTERPERSONAL
Evidence based information (knowledge transfer bottom-up)
Training in risk/communication of risk
Training in communication skills and motivational interviews
INTRAPERSONAL
Motivation of the practice manager and center staff
Health literacy strategies
Tailored interventions based on patients' social and cultural priorities
Team building within the PCHC (role clarification)
Coordination with specialized care (stepped care)
INSTITUTIONAL
Protocol guides adapted to the characteristics of the center and area
PP&HP approach strategies (“The X trimester”; Alarms/reminders)
Self-management of agenda by professionals
Self-management of PC center resources
COMMUNITY
Coordination of PC professionals with formal and informal community resources available (social prescribing)
Inclusion of PP&HP, biopsychosocial model and person-centered care in university education.
Mass media campaigns (social marketing) to inform the population of the importance of PP&HP activities and what they can expect from the health system.
Control of mass media campaigns and the impact of the pharmaceutical industry on activities that run against healthy living habits (e.g., smoking)
POLICY
Higher investment in primary care and PP&HP
Promotion of community and social resources (integrated care).
Inform policy makers about the benefits of preventive activities (Knowledge transfer top-down)

One of the main factors affecting the implementation of PP&HP activities is related to the beliefs, attitudes and motivations of professionals. According to the theory of planned behavior [56], primary care professionals' intention to implement PP&HP depends on the professionals' attitude toward PP&HP, subjective norms and the professionals' perceived control over the implementation of these activities. Erroneous beliefs about PP&HP activity effectiveness can easily be corrected by generating a rich body of evidence and using it to support the promotion of the activities. To achieve a change in the beliefs, attitudes and motivations of professionals, it is essential that there is adequate knowledge transfer from the scientific community to, on the one hand, policy-makers so that they can conduct a top-down transfer and, on the other, to clinicians who can provide a complementary bottom-up approach [57]. In addition, the skills required to carry out PP&HP activities should be included in health professionals' training in university education and subsequent continuous training, moving from a biomedical to a biopsychosocial model of care. This would be useful on two levels: providing the necessary skills (i.e. for risk assessment and motivational interview) and reinforcing the professionals' self-concept. This will impact in the perceived control over the implementation of PP&HP and in the intention to implement it [56]. The policies must incentivize PP&HP at different levels, motivating managers whose teams will carry out the implementation and launching health education and social marketing campaigns with the aim of increasing social awareness of the importance of PP&HP in health care. In addition to facilitate the development of primary prevention and health promotion activities by reducing the side-effects of PP&HP on the patient-physician relationship, if professionals perceive that managers and patients want them to implement PP&HP (positive subjective norm), they would present higher motivation to do it [56]. At a more basic level, the health center would need to build well-coordinated teams where members have clearly defined roles in relation to PP&HP. Managers will need to facilitate self-management with respect to professionals' agendas so that they can adapt to timetable changes and patient follow-up.

Activities should be tailored and adapted to the PC context as well as to the social, cultural and community context of each area where implementation takes place to encourage the acceptability, feasibility and sustainability of the interventions/activities [58]. In this way, the problem of adaptation to health recommendations and clinical practice guidelines in the real PC context and the community where they are implemented can be solved, changing the negative attitudes of GPs and nurses to guidelines. The mechanisms though which the factors affecting PP&HP activities are generated can differ between public and private systems. This also needs to be taken into account.

To maintain awareness of the sociocultural context, it is important to facilitate the creation of teams within the PC center, as well as professional training and adaptation to the recommendations made at the health center itself. This is related to patient-centered health care, with comprehensive care and health care continuity [59]. It is important that policies promote integrated care between formal and informal community and health system resources [60]. Thus, it is crucial that the PC center is in contact with community social resources (e.g., gymnasiums, pharmacies, associations, schools) to coordinate the use of these resources and reach agreement on activity protocols with all interested parties. These resources should be included in the adapted guides in each of the centers. Within the health system, the coordination of health services should be improved along with communication channels to avoid sending contradictory messages on PP&HP.

Useful tools may include the use of assessment campaigns (e.g., the alcohol trimester, the exercise trimester) which could provide professionals with the excuse to deal with issues that could be perceived as delicate. The use of reminders in computerized clinical histories is, in theory, a good strategy although their real effectiveness will be conditioned by the attitude of the professional; too many tools could overwhelm the professional.

The informants in some of the studies identified in the search represented professional groups other than GPs and nurses like in the study by Blumenthal 2007 [61] (dietitians, administrators, social workers and pharmacists) or Ribera 2006 [62] (politicians, researchers, academics, representatives of family medicine associations, physical activity professionals and reporters). These studies were excluded because the specific discourse of the GPs and nurses could not be discerned. However, these studies noted the importance in PP&HP activities of other professionals within the PCHC (such as health workers or health assistants) or even from outside the PCHC (i.e. politicians or pharmacists). The inclusion of these other categories of professionals could alleviate the workload of the GPs and nurses.

### Implications for Research

As this review shows, there is a great deal of information on what are referred to as the barriers and facilitators which affect the implementation of PP&HP activities in PC from the perspective of the physicians and nurses. However, the majority of these studies have not taken into account the fact that the PC focus is comprehensive and multifactorial and there is not much information on barriers in relation to PP&HP aimed at multi-risk management. In only one of the studies identified was this problem tackled [36]. Further research needs to be conducted to assess this issue.

The results of this synthesis should be complemented with a synthesis on the barriers and facilitators in PP&HP from the point of view of the patients who would receive the interventions and any other professionals who may be involved.

Our review has revealed that there are certain deficiencies, at least with respect to reporting the methodology employed in the qualitative studies on this issue. As mentioned previously, most studies do not describe the researchers' theoretical focus, the sociocultural context, sampling methods or the analysis, while details available on measures taken to ensure rigor are scarce. This could be due to limited space in biomedical journals where these types of studies are typically published.

However, regarding qualitative synthesis of results, it has been suggested that ‘inclusion of poor quality studies is unlikely to have a very distorting impact on qualitative synthesis’ [63].

With respect to the implications for practice that result from this study, it is important to assess the effectiveness of the recommendations described.

### Strengths and Limitations

To the best of our knowledge, this is the first attempt to synthesize all the available evidence regarding factors affecting PP&HP implementation in PC from the professionals' perspective. The strengths of this meta-ethnographic synthesis lie in the extensive literature search. Moreover, the inclusion of papers detailing different theoretical approaches provided in-depth insight into the study topic. A multi-disciplinary team enriched the results of the synthesis as they were able to provide various re-interpretations of the findings. At least two researchers participated independently at every step of the synthesis and then triangulated the results. This synthesis was also externally audited by both a group of qualitative researchers and a multidisciplinary team of primary care professionals from different Spanish regions. These increased the credibility, consistency and confirmability of the results of the synthesis [15], [64].

Regarding limitations, the synthesis only took into account the views of physicians and nurses. These are the main players in the implementation of PP&HP activities in PC. However, we excluded the perspective of other professionals in PCHC as well as those of the patient and community. This needs to be addressed in future research as stated above.

Finally, we may have missed relevant information as we only searched 3 electronic databases, we only included English and Spanish studies and we did not search gray literature. However, the electronic search was extensive and complemented by hand-searches and advice from experts in the field. The amount of information retrieved was considerable and enough to saturate the information.

## Conclusions

We have carried out a global qualitative synthesis on PP&HP from the perspective of physicians and nurses that can be applied to any context and any of the PP&HP activities. This review takes into account the different levels (Fig. 2) from the perspective of the professionals and how these levels are inter-related. A lack of research on barriers and facilitators has been detected in the implementation of PP&HP activities in multi-risk management.

Moreover, the conceptual overview provided by the synthesis resulted in the development of some practical recommendations for the design of PP&HP in PC. However, the effectiveness of these recommendations needs to be demonstrated.

## Supporting Information

Table S1 Detailed search strategies in electronic databases.
(DOC)
