# Supplementary material for: Barriers and Facilitators for the Implementation of Primary Prevention and Health Promotion Activities in Primary Care: A Synthesis through Meta-Ethnography
Source: PLoS One. 2014 Feb 28;9(2):e89554. doi: 10.1371/journal.pone.0089554 (PMC3938494; doi:10.1371/journal.pone.0089554)
Supplement: Table S1 — Detailed search strategies in electronic databases. (DOC) [file pone.0089554.s001.doc]

**Table S1**. Detailed search strategies in electronic databases

| Pubmed searches (15/10/2012) | | | |
| --- | --- | --- | --- |
| Researcher 1 | | ("Qualitative Research"[Mesh] OR "qualitative study" OR "qualitative research") AND ("Primary Care Nursing"[Mesh] OR "Physicians, Primary Care"[Mesh] OR "General practitioner" OR "Family physician" OR "Primary care physician" OR "General practice physician" OR "Primary care nurse" OR (nurse AND ("primary care*" OR "general practice" ))) AND ("Preventive Health Services"[Mesh] OR "Preventive Health Services" OR "Preventive Medicine" OR "Primary prevention" OR "Early Intervention" OR "Early Medical Intervention" OR "Health Promotion“) | |
| Researcher 2 | | (beliefs OR attitude* OR opinion* OR perception* OR barriers OR facilitators) AND ("General Practitioners"[Mesh] OR "Nurses"[Mesh] OR "general practitioners" OR "primary care physician" OR "nurse") AND ("Primary Prevention"[Mesh] OR "Health Promotion"[Mesh] OR "primary prevention" OR " health promotion" ) AND ("Qualitative Research"[Mesh] OR "qualitative“) | |
| Sensitivity search | | ((("Dyslipidemias"[Mesh] OR "Dyslipidemias" OR "Dyslipidemia" OR "Hypercholesterolemia") OR ("Hypertension"[Mesh] OR "Hypertension" OR "High Blood Pressure" OR "High Blood Pressures") OR ("Risk Reduction Behavior"[Mesh] OR "Risk Reduction Behaviour" OR "Lifestyle Risk Reduction" OR "Lifestyle Risk" OR "Risk Behaviour" OR "Risk Reduction")) NOT (("Primary Prevention"[Mesh] OR "Health Promotion"[Mesh] OR "primary prevention" OR " health promotion" ) OR ("Preventive Health Services"[Mesh] OR "Preventive Health Services" OR "Preventive Medicine" OR "Primary prevention" OR "Early Intervention" OR "Early Medical Intervention" OR "Health Promotion“))) AND (("General Practitioners"[Mesh] OR "Nurses"[Mesh] OR "general practitioners" OR "primary care physician" OR "nurse" OR "Primary Care Nursing"[Mesh] OR "Physicians, Primary Care"[Mesh] OR "General practitioner" OR "Family physician" OR "Primary care physician" OR "General practice physician" OR "Primary care nurse" OR (nurse AND ("primary care*" OR "general practice" )))) AND ((beliefs OR attitude* OR opinion* OR perception* OR barriers OR facilitators OR "Qualitative Research"[Mesh] OR "qualitative study" OR "qualitative research")) | |
| ISI Web of Knowledge (22/01/2013) | | | |
| Researcher 1 | Topic=((("qualitative study" OR "qualitative research") AND ("Primary Care Nursing" OR "Primary Care Physicians" OR "General practitioner" OR "Family physician" OR "Primary care physician" OR "General practice physician" OR "Primary care nurse" OR (nurse AND ("primary care" OR "general practice" ))) AND ("Preventive Health Services" OR "Preventive Medicine" OR "Primary prevention" OR "Early Intervention" OR "Early Medical Intervention" OR "Health Promotion"))) Timespan=All Years. Search language=English | | |
| Researcher 2 | Topic=((beliefs OR attitude* OR opinion* OR perception* OR barriers OR facilitators) AND ( "general practitioner*" OR "primary care physician" OR "nurse*") AND (“primary prevention" OR " health promotion" ) AND ("Qualitative Research" OR "qualitative")) Languages=( ENGLISH OR SPANISH ) Timespan=All Years. | | |
| CINHAL (28/01/2013) | | | |
| Researcher 1: | | | AB ( "qualitative study" OR "qualitative research" ) AND TX ( "Primary Care Nursing" OR "Primary Care Physicians" OR "General practitioner" OR "Family physician" OR "Primary care physician" OR "General practice physician" OR "Primary care nurse" OR (nurse AND ("primary care" OR "general practice" )) ) AND TX ( "Preventive Health Services" OR "Preventive Medicine" OR "Primary prevention" OR "Early Intervention" OR "Early Medical Intervention" OR "Health Promotion") |
| Researcher 2 | | | AB ( "Qualitative Research" OR "qualitative" ) AND AB ( beliefs OR attitude* OR opinion* OR perception* OR barrier* OR facilitator* ) AND TX ( "general practitioner*" OR "primary care physician" OR "nurse*" ) AND TX ( “primary prevention" OR " health promotion" ) |
